# Maternal supplementation with chia oil attenuates hepatic metabolic disturbances in mice subjected to postnatal undernutrition

**DOI:** 10.3389/fnut.2025.1636396

**Published:** 2025-08-20

**Authors:** Estéfany Ribeiro Leão, Isaac Konig, Sarah Melo Silva Marques, Geraldo de Sousa Candido, Mary Suzan Varaschin, Laura Cristina Jardim Pôrto Pimenta, Isabela Coelho de Castro

**Affiliations:** ^1^Department of Nutrition, Federal University of Lavras, Lavras, Brazil; ^2^Department of Chemistry, Federal University of Mato Grosso, Cuiabá, Brazil; ^3^Department of Veterinary Medicine, Federal University of Lavras, Lavras, Brazil

**Keywords:** alpha-linolenic acid, fetal development, oxidative stress, omega-3, malnutrition

## Abstract

**Background:**

Early postnatal undernutrition, leading to impaired growth and low body weight, has been associated with enduring metabolic alterations that may persist into adulthood. We proposed that plant-based ω-3 fatty acids, as in maternal supplementation, attenuate metabolic alterations induced by postnatal dietary restriction, such as glucose disturbances and oxidative stress.

**Methods:**

To test this, we investigated the effects of maternal supplementation with two distinct doses of Chia Oil (ChO) (2.5 or 5 g/kg body mass) on metabolic parameters in BALB/c mice subjected to postnatal undernutrition. The undernutrition model was created by increasing the litter size to 15–16 pups, forming the undernutrition (UN) group. These UN groups received maternal ChO supplementation at 2.5 g/kg or 5 g/kg b.m., labeled as UN2.5 and UN5, respectively.

**Results:**

By day 21, the UN5 group showed less weight gain compared to the UN2.5 group. At 120 days, glucose tolerance tests revealed a lower area under the curve in both supplemented groups compared to the UN animals. A maternal dose of 5 g/kg b.m. of ChO was linked to more favorable oxidative stress markers, suggesting this effect is not due to changes in antioxidant enzymes like superoxide dismutase and catalase, which remained stable in the liver tissue in this model. This dose provided a slight benefit in reducing metabolic changes, with the UN5 group showing lower total hepatic lipid levels. Additionally, histopathological analysis of the tissue revealed no alterations in the experimental groups.

**Conclusion:**

These observations suggest a protective role of maternal ChO supplementation at a dose of 5 g/kg b.w. against metabolic impairments induced by postnatal undernutrition.

## 1 Introduction

Barker (1), in his epidemiological study, was the pioneer in suggesting that occurrences during early human development might significantly influence the onset and advancement of long-term illnesses later in life. He demonstrated that infants with low birth weight, resulting from maternal undernutrition during pregnancy, are more likely to develop obesity and cardiometabolic disorders as adults (1).

Therefore, changes in nutrition and growth during the early postnatal period can have a lasting impact on metabolism in later years (2, 3). The composition and amount of milk consumed during suckling, which varies according to the maternal diet, can cause long-lasting metabolic alterations that lead to the development of obesity (4, 5). Consequently, imbalances in nutrient supply, whether insufficient or excessive, during the prenatal and perinatal stages can predispose individuals to obesity and metabolic disorders in adulthood. Manipulating litter size has been used to simulate experimental under- or overnutrition in rodents (5–8).

Animals raised in large litters experience alterations in leptin and ghrelin secretion and changes in energetic metabolism, including reduced expression of glucose transporters in cardiac and skeletal muscles and increased insulin resistance in key organs controlling glucose homeostasis, such as the liver, muscle, and adipose tissue (9–12). Undernutrition during the nursing phase can disrupt the oxidative balance in offspring, leading to elevated lipid peroxidation and reduced activities of superoxide dismutase (SOD), glutathione peroxidase, and catalase (CAT). This indicates that these enzymes are particularly susceptible to oxidative stress due to larger litter size. Nonetheless, the specific mechanisms driving these changes are still unclear (13, 14).

Maternal nutrition during gestation and lactation plays a key role in the metabolic programming of offspring. Moreover, the quantity and quality of maternal dietary fat intake have profound health implications during and after pregnancy. Polyunsaturated fatty acids (PUFA) are essential for fetal growth and development; however, high consumption of Omega-6 (PUFA ω-6) is associated with an increased incidence of complications at birth and in adulthood (11, 14).

During pregnancy, the only source of PUFAs for the developing fetus is the mother, through placental transfer. Maternal intake of fatty acids during gestation and their subsequent transfer to the fetus are essential for fetal growth and development (15–17).

Maternal consumption of omega-3 polyunsaturated fatty acids (PUFA ω-3) has demonstrated beneficial effects on the development of the fetal neurological and immune systems. Such intake may help prevent obesity, insulin resistance, and cardiovascular disorders later in life (14, 17). Plants serve as a key nutritional source of alpha-linolenic acid (ALA), the precursor of PUFA ω-3, which undergoes elongation to produce ω-3 eicosapentaenoic acid (EPA) and docosahexaenoic acid (DHA) (18). In this regard, chia seeds have recently attracted considerable interest due to their high ALA content (55%−66%), underscoring their potential advantages for human health and nutrition.

Furthermore, they are important sources of protein, dietary fiber, minerals (including iron and calcium), and bioactive compounds (such as tocopherols and phenolic compounds), increasing their potential benefits to human health (19, 20). Chia oil has been reported to improve lipid profiles by increasing HDL-c levels and reducing total cholesterol. Additionally, it contributes to glycemic homeostasis by enhancing glucose tolerance and insulin sensitivity. Moreover, chia oil increases the activity of antioxidant enzymes, including superoxide dismutase (SOD), catalase (CAT), glutathione peroxidase (GPx), and glutathione reductase (GR) (21).

Much research has focused on the association between malnutrition and specific micronutrient deficiencies, while there are little data on essential fatty acids in children with severe malnutrition (22). We hypothesized that plant-based ω-3 fatty acids, as in maternal supplementation, attenuate metabolic alterations induced by postnatal dietary restriction, such as glucose disturbances and oxidative stress. Thus, our study focused on evaluating the effects of maternal supplementation with plant-based ω-3 fatty acids (ALA) via chia oil on attenuating metabolic dysfunctions, such as glucose imbalance and oxidative stress, induced by postnatal undernutrition in rat offspring. To test this hypothesis, we evaluated the role of maternal chia oil supplementation at two dosages in mitigating metabolic abnormalities in undernourished BALB/c mice and identified the optimal dose of chia oil.

## 2 Materials and methods

### 2.1 Phytochemical analysis of the chia oil (GC/FID)

The chia oil used in this study was purchased from a local market (Pazze, 05071-6). Phytochemical analysis of chia oil (ChO) was conducted at the Central Laboratory for Chemical Analysis and Prospecting of the Federal University of Lavras. Commercially available Chia Oil (Pazze, 05071-6, Panambi/RS, Brazil) was used. Fatty acid samples were analyzed using gas chromatography (GC 2010-Shimadzu) equipped with a flame ionization detector and split injector at a split ratio of 1:50. A 100 m long, 0.25 mm diameter, and 0.2 μm thick Supelco film-fused silica capillary column (SP-2560; Bellefonte, PA, USA) was used. The chromatographic conditions consisted of an initial column temperature of 140 °C held for 5 min, followed by a temperature ramp of 4 °C/min until reaching 240 °C, which was maintained for 30 min, resulting in a total run time of 60 min. Both the injector and detector were set at 260 °C. Helium was used as the carrier gas. Fatty acids were identified by comparing their retention times with those of the chromatographic standards (Supelco 37 standard FAME Mix, Supelco Inc., USA). The results were expressed as the percentage of total fatty acids detected.

### 2.2 Animals

The experimental protocol involving animals was reviewed and approved by the local Animal Ethics Committee (CEUA/UFLA/050/2019). Thirty BALB/c mice, comprising twenty-two females and eight males aged between 45 and 55 days, were sourced from the Animal Care Facility at the Federal University of Lavras (UFLA). All animals were maintained under conditions that complied with the ethical standards established by our institution. The BALB/c mice had free access to water and a standard rodent diet in pellet format (23) (Table 1; Nuvilab CR-1, Quimtia S/A, Colombo, PR, Brazil).

**Table 1:** Nutritional composition of the normocaloric Nuvilab Cr-1 commercial chow.

**Component**	**Amount (g/kg)**	**Energy (kcal/kg)**
**Carbohydrates**		
Corn starch (S.Q.)	530	2,120
Cellulose	80	–
**Proteins**		
Casein	220	880
L-cystine	3	-
**Fats and extracts**		
Soybean oil	70	630
Ether extract	40	360
**Fibers**		
Brute fiber	50	–
**Micronutrients**		
Mineral mix	35	–
Vitamin mix	10	–
Choline	2.5	–
Total	1,000	3,990

S.Q., sufficient quantity. Nutritional information and ingredient composition were based on the formulation of the Nuvilab CR-1 irradiated feed, manufactured by Quimtia (Curitiba, Brazil). Data based from da Silva Monteiro et al. (23).

### 2.3 Maternal supplementation

Following a review of the main findings of chia oil supplementation in animal models (21), it was concluded that there is no consensus in the literature regarding the optimal dose for preclinical investigations. Therefore, two doses (2.5 and 5 g/kg body mass) were selected for evaluation in this study based on the recommended intake of ALA, which is reported to be 1.1–2.2 g/day (24). The mice were allocated into three experimental groups: two groups received different doses of chia oil supplementation, and the third group served as an unsupplemented control group. Female mice were administered chia oil via oral gavage at doses of 2.5 g/kg or 5 g/kg body weight, starting from mating and continuing until the offspring were weaned at 21 days of age. The total duration of supplementation ranged from 38 to 42 days. All animals were fed ad libitum with the same diet (53% carbohydrate, 7% fat, 22% protein), as described in Table 1. Feeding restriction occurred only during the nursing period by increasing the litter size, as detailed in the section below.

### 2.4 Experimental design

Female mice were housed in groups of three animals per cage under controlled environmental conditions. One male was introduced into each cage for mating and remained with the females for 14 days, which was the time needed to ensure all females were pregnant. After this period, the males were removed, and the females remained together until the offspring were born. On postnatal day 3, the litters were reassigned to their respective experimental groups. To induce early postnatal undernutrition, as described by Caron et al. (25), the original litter was increased to 15–16 pups (females and males). Litters of this size were assigned to the Undernutrition (UN) and Undernutrition + Chia Oil supplementation (2.5 g/kg or 5 g/kg body mass; UN2.5 and UN5, respectively) groups. The Control group (C) litters contained 8–10 pups (females and males).

**Figure 1:**
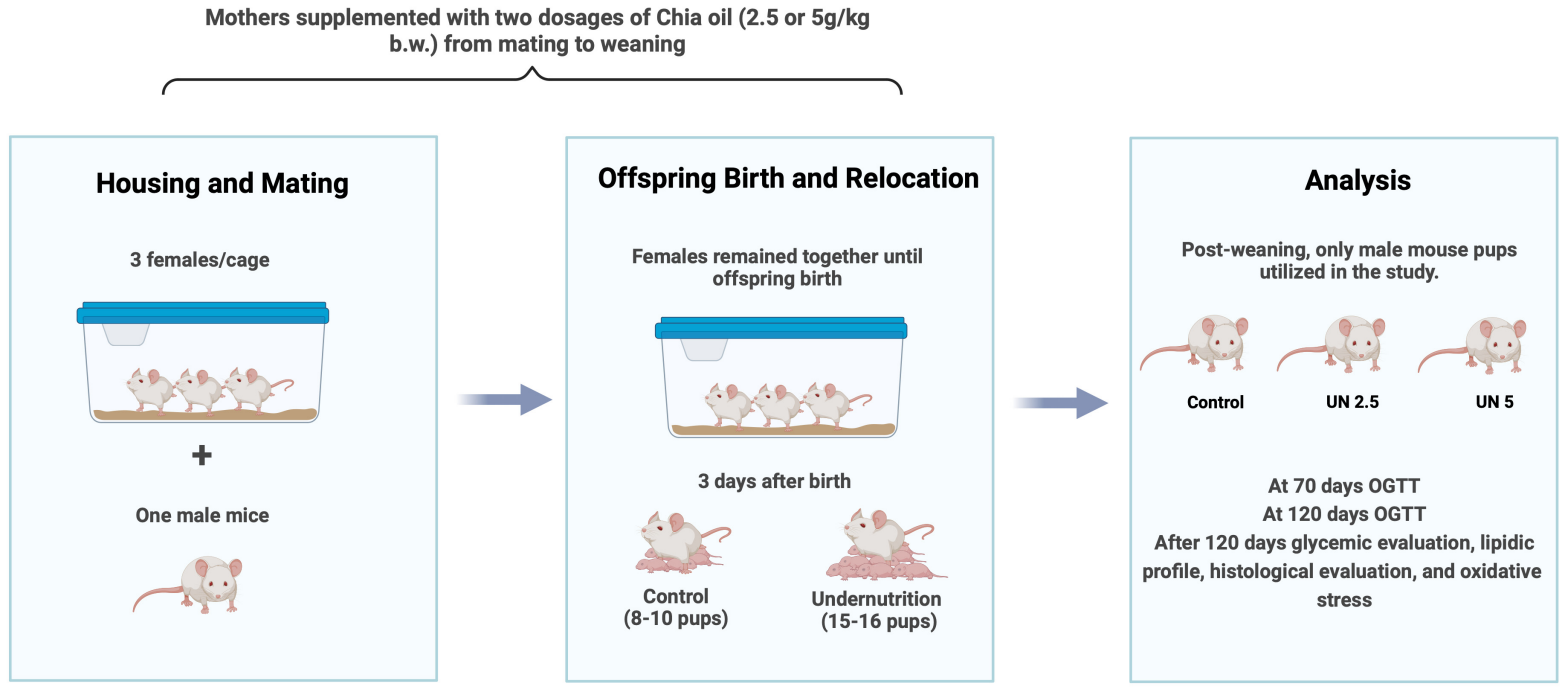
Experimental design. Pregnant female mice were housed in groups of three, each paired with a male for mating, and which the males were removed. Following birth, the litter sizes were adjusted based on the experimental group requirements. Only male pups were used in the study. The oral glucose tolerance test was conducted at 70 and 120 days post-birth to assess glucose levels. Additionally, lipid profiles, histological parameters, and oxidative stress were evaluated. C, control; UN, undernutrition group; UN2.5, undernutrition group with chia oil (2.5 g/kg b.m.) supplementation; UN5, undernutrition group with chia oil (5 g/kg b.m.) supplementation; b.m., body mass.

In this study, only male pups were used. Therefore, the experimental procedure was repeated as necessary to meet the minimum statistical requirements for each group. After weaning, males were separated from females and housed in groups of five in each cage. An overview of the experimental design is presented in Figure 1.

Body weights were recorded at 21, 70, and 120 days of age, and food consumption was monitored weekly, beginning on day 21. The percentage of body mass gain was calculated using the following formula: (*FW*−*IW*) × 100, FW represents the final weight (g) at 120 days, and IW represents the initial weight (g) at 21 days. The Lee index was calculated using the following formula: [$\sqrt[{3}]{weight\;\left(g\right)/nose-to-anus\;length\;\left(cm\right)}$] (26).

At 121 days of age, the animals underwent a 12-h fasting period before being anesthetized with a combination of ketamine (200 mg/kg body weight), xylazine (15 mg/kg body weight), and inhaled isoflurane. Euthanasia was performed via cardiac exsanguination. Samples of epididymal, retroperitoneal, and liver tissues were collected, weighed, and stored at −80 °C. A portion of the liver was preserved in formaldehyde for histological analysis. Blood samples were drawn for analysis, and the plasma was separated and frozen at −80 °C for later use.

PND70 and PND120 were chosen to evaluate outcomes during early and established adulthood in mice, respectively (27).

### 2.5 Assessment of glucose tolerance

An oral glucose tolerance test (OGTT) was performed on mice at 70 and 120 days of age, as described previously. The animals were fasted overnight for 12 h with unrestricted access to water *ad libitum*. Glucose was administered via oral gavage at a dose of 2 g/kg body mass, and blood samples were collected from the tail vein at 0, 30, 60, 90, and 120 min after the administration. Blood glucose concentrations were measured using an Accu-Chek^®^ glucometer (Roche Diagnostics, Indianapolis, IN, USA) and expressed in mmol/L. The area under the curve (AUC) was determined using the trapezoidal method based on glucose concentrations over time.

### 2.6 Analysis of glycemic and lipid parameters

Fasting glucose, triglyceride, and total cholesterol levels were quantified using colorimetric assay kits (Labtest, Brazil). The results are presented in mmol/L.

### 2.7 Histological preparation and analysis

Liver tissue fragments were collected for histological analysis and fixed in 4% formaldehyde solution for 72 h. The samples were then dehydrated using serial ethanol concentrations (70%, 80%, 90%, and 100%) and embedded in paraffin. The material was sectioned using a microtome (Lupetec MRP09) to a thickness of 5 μm and distributed on three glass slides for each sample. The sections were stained with hematoxylin and eosin for the general analysis of liver morphology. The sections were analyzed using an Olympus CBA light microscope to evaluate the presence of histopathological changes.

### 2.8 Determination of hepatic lipid content

Hepatic lipids were extracted using organic solvents as described by Folch et al. (28). For the assay, the lipid extracts were incubated at 37 °C overnight for complete drying. The total hepatic lipid content was measured. The dried lipids were then solubilized in 500 μl of isopropanol. Total cholesterol and triglyceride levels were quantified using commercially available colorimetric kits (Labtest, São Paulo, Brazil).

### 2.9 Evaluation of oxidative markers in liver and epididymal adipose tissue

Liver and epididymal adipose tissue samples (100 mg per animal) were homogenized in phosphate-buffered saline (PBS). The resulting homogenates were centrifuged at 7250 × g for 10 min at 4 °C. The supernatants were collected and stored at −20 °C for further analysis. Total protein content was determined to normalize the data using the Bradford method (29) for the analyses described below.

#### 2.9.1 Peroxidation assay

Lipid peroxidation was determined using the Thiobarbituric Acid Reactive Substances (TBARS) technique according to Wallin et al. (30), by adding 0.5 ml TBARS solution (15 g trichloroacetic acid and 0.375 g thiobarbituric acid) to 6.25 ml of 4.0 M HCl. The samples were incubated at 100 °C for 15 min and then cooled to room temperature. Following the addition of 0.75 ml of butanol, the mixture was centrifuged, and the absorbance was recorded at 535 nm. TBARS levels were expressed as nanomoles of malondialdehyde (MDA) per milligram of total protein.

#### 2.9.2 Hydroperoxide assay

Hydroperoxide concentrations were measured as described by Banerjee et al. (31). Briefly, hydroperoxides oxidize ferrous ions (Fe^2+^) to ferric ions (Fe^2+^) in an acidic medium. The resulting ferric ions then interact with xylenol orange in the reagent to form a colored complex. Absorbance was measured at 550 nm. The results are expressed as μmol/mg of protein.

#### 2.9.3 Catalase activity assay

Catalase (CAT) activity was measured as described by Aebi (32). Catalase activity was assessed by monitoring the decrease in absorbance due to H_2_O_2_ consumption at 240 nm. Each reaction mixture contained 100 μl of the sample, 2000 μl of PBS, and 50 μl of 0.3 M H_2_O_2_. Enzyme activity was calculated using the following equation: (abs0sec – abs60sec/0.1) × dilution factor/mg protein, where abs0seg is the initial absorbance and abs60seg is the final absorbance. The results are expressed as protein concentration (mg/ml).

#### 2.9.4 Superoxide dismutase activity

Superoxide dismutase (SOD) activity was determined based on its ability to inhibit auto-oxidation, measured at an absorbance of 550 nm (33). The assay relies on superoxide generation through pyrogallol autoxidation and the subsequent inhibition of the superoxide-mediated reduction of the tetrazolium salt MTT [3-(4,5-dimethyl-thiazol-2-yl)-2,5-diphenyl tetrazolium bromide]. The reaction was terminated by adding 150 μl dimethyl sulfoxide (DMSO). The results were expressed as SOD units per milligram of protein per milliliter (U SOD/mg protein/ml).

### 2.10 Statistical analysis

Statistical analyses were performed using GraphPad Prism software (version 8.0). Prior to the analysis, the normality of the data distribution was assessed using the Shapiro–Wilk test. Group differences were analyzed using one-way analysis of variance (ANOVA), followed by Bonferroni *post hoc* comparisons when appropriate. Results are presented as mean ± standard error of the mean (S.E.M.), and statistical significance was defined as *p* < 0.05.

## 3 Results

### 3.1 Chemical characterization of chia oil

Chromatographic analysis of chia oil composition revealed that linolenic acid is the predominant biocomponent, accounting for 52.3% of the oil. The other significant components include linoleic acid (22.7%), oleic acid (13.4%), palmitic acid (7.2%), and stearic acid (3.7%), as well as various other fatty acids which are present in smaller quantities (Table 2). The chemical components of chia oil found in this study were similar to those reported in other studies (34). However, other studies suggest that the quantities of these compounds are slightly different from those observed in this study (35, 36).

**Table 2:** Content of fatty acids in chia oil. Fatty acids are expressed as equivalent milligrams of each compound per 100g of chia oil (g/100g).

**Fatty acids (g/100g)**	**Chia oil**
Linolenic acid	52.3
Linoleic acid	22.7
Oleic acid	13.4
Palmitic acid	7.2
Stearic acid	3.7
cis-8,11,14 Eicosatrienoic acid	0.16
Arachidic acid	0.15
Eicosapentaenoic acid	0.09
**∑fatty acids**	**99.7**

∑ fatty acids: total amount of all measured fatty acids.

These variations in composition can be explained by factors such as the geographical region where the crop is grown, species type, physical and chemical characteristics of the soil, time of harvest, plant age, and extraction methods used (37).

### 3.2 Body parameters

**Figure 2:**
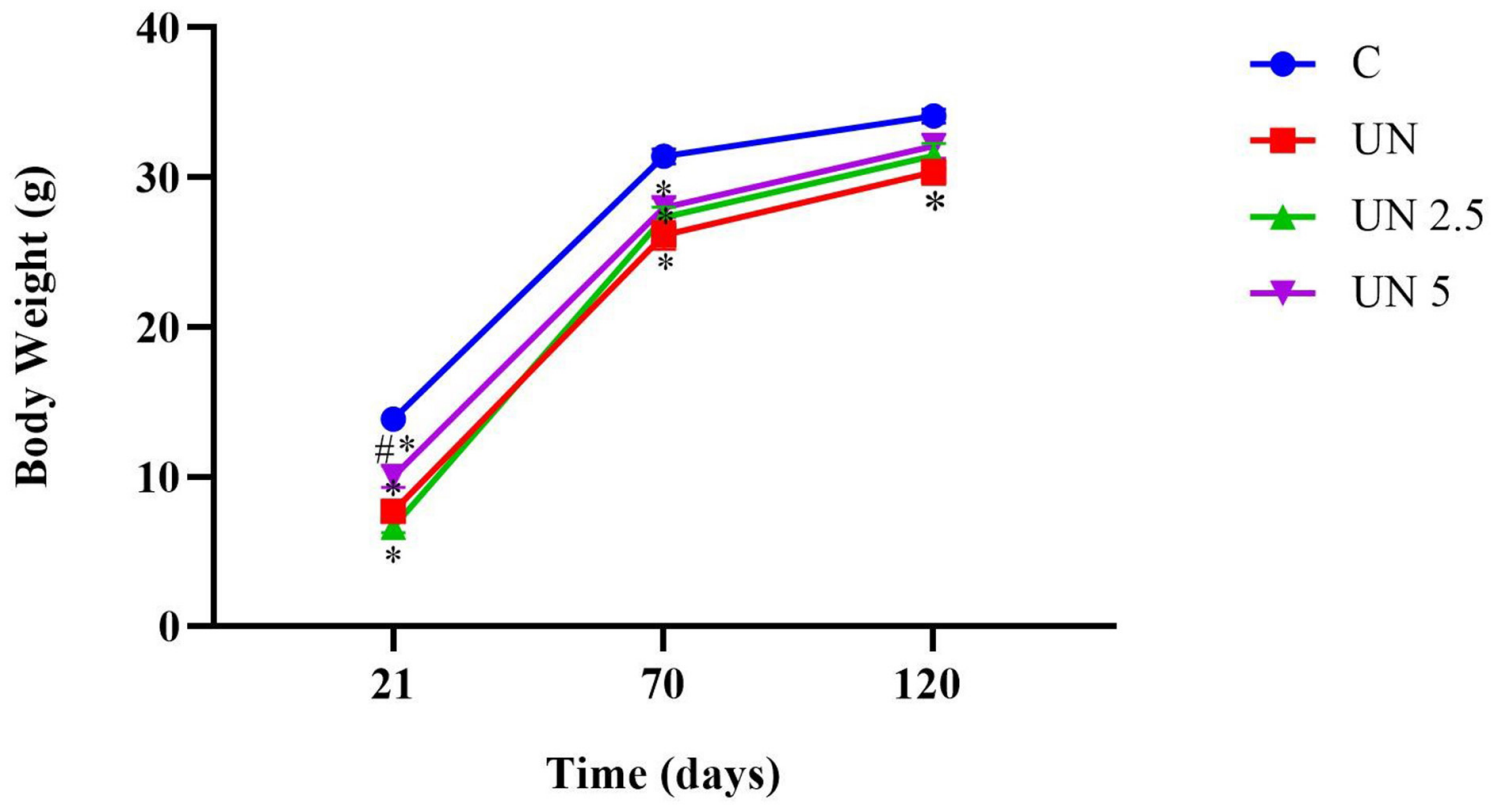
Postnatal undernutrition leads to a lower body mass than the control litter at 21, 70, and 120 days. Data are presented as mean ± standard error of the mean. *p < 0.05 vs. C. ^#^*p* < 0.05 vs. UN2.5. Data were analyzed using one-way analysis of variance, followed by Bonferroni *post hoc* test. Control (C, *n* = 9), undernutrition (UN, *n* = 5), undernutrition with 2.5 g/kg b.m. chia oil supplementation (UN2.5, *n* = 8), and undernutrition with 5 g/kg b.m. Chia oil supplementation (UN5, *n* = 10).

Body weights were recorded at 21 and 70 days of age. At both time points, all undernutrition groups (UN, UN2.5, and UN5) exhibited significantly lower body mass than the control group (*p* < 0.05). Notably, at 21 days, the UN5 group showed higher body weight than the UN2.5 group (*p* < 0.05). By 120 days, only the UN group continued to display reduced body mass relative to the controls (*p* < 0.05). The trajectory of body mass over time is shown in Figure 2.

Although slight differences in weight were observed at specific time points, overall body mass gain was higher in the UN, UN2.5, and UN5 groups than in the control group. Within the undernourished groups, the UN2.5 group exhibited significantly greater weight gain than the UN5 group (*p* < 0.05). No significant differences in food intake were observed among the experimental groups. Similarly, the Lee index remained unchanged across all groups.

All organ and tissue weights were expressed relative to the body mass of each mouse. Epididymal adipose tissue weight was significantly reduced in all undernutrition groups (UN, UN2.5, and UN5) compared to the control group (*p* < 0.05). Similarly, retroperitoneal adipose tissue weights were also lower in these groups than in the control group (*p* < 0.05), as shown in Table 3. In contrast, liver weight was significantly greater in the UN, UN2.5, and UN5 groups compared to the Control group (*p* < 0.05).

**Table 3:** Effect of maternal supplementation with chia oil on body parameters in mice from litter control and undernourished.

**Body parameters**	**C**	**UN**	**UN2.5**	**UN5**
BM gain (%)	147.9 ± 7.38	306.0 ± 37.6^*^	377.9 ± 29.1^*^	242.2 ± 34.17^*^^&^
Lee index at 120 days	1.13 ± 0.01	1.06 ± 0.02	1.08 ± 0.03	1.09 ± 0.03
EAT weight (mg/g b.m.)	16.32 ± 0.95	8.52 ± 1.19^*^	8.87 ± 1.07^*^	8.03 ± 1.35^***^
Liver weight (mg/g b.m.)	38.38 ± 0.6	41.15 ± 0.8^*^	40.82 ± 0.6^*^	47.76 ± 2.9^*^
Retroperitoneal adipose tissue weight (mg/g b.m.)	3.35 ± 0.2	1.81 ± 0.3^*^	2.10 ± 0.3^*^	1.24 ± 0.2^***^

BM, body mass; EAT, epididymal adipose tissue. Values are presented as the mean ± SEM. ^*^p < 0.05 and ^***^p < 0.0001 vs. Control; ^&^*p* < 0.05 vs. UN2.5. The data were analyzed using a one-way analysis of variance followed by the Bonferroni post hoc test. Control (C, *n* = 9), Undernutrition (UN, *n* = 5), Undernutrition with 2.5 g/kg b.m. Chia oil supplementation (UN2.5, *n* = 8), and Undernutrition with 5 g/kg b.m. Chia oil supplementation (UN5, *n* = 10).

### 3.3 Metabolic parameters

The fasting glucose and lipid profiles of the experimental groups are shown in Table 4. The UN5 group exhibited greater fasting glucose levels than the control group (*p* < 0.05), but no significant difference was observed compared to the UN group. No significant differences were found among the groups regarding plasma triglyceride and total cholesterol levels. Similarly, no significant differences were detected in hepatic cholesterol and triglyceride levels between the groups. Notably, the UN5 group presented reduced total hepatic lipid content compared to the Control, UN, and UN2.5 groups (*p* < 0.05).

**Table 4:** Effect of maternal supplementation with chia oil on metabolic parameters in mice from litter control and undernourished.

**Metabolic parameters**	**C**	**UN**	**UN2.5**	**UN5**
**Blood plasma**				
Fasting glucose (mmol/L)	3.7 ± 0.3	5.5 ± 1.2	5.0 ± 0.7	6.8 ± 0.6^*^
Triglycerides (mmol/L)	0.7 ± 0.1	0.9 ± 0.2	0.7 ± 0.1	1.2 ± 0.2
Total cholesterol (mmol/L)	1.6 ± 0.2	2.0 ± 0.3	1.7 ± 0.3	2.0 ± 0.4
**Hepatic lipids**				
Total lipids (mg of lipids/g of liver)	64.1 ± 3.0	55.7 ± 6.4	59.1 ± 7.5	27.8 ± 6.9^*^^#&^
Hepatic cholesterol (mmol/L)	3.8 ± 0.4	3.7 ± 0.1	3.8 ± 0.6	3.8 ± 1.0
Hepatic triglycerides (mmol/L)	1.9 ± 0.2	1.8 ± 0.3	2.6 ± 0.1	1.4 ± 0.2

Values are presented as mean ± SEM. ^*^p < 0.05 vs. control; ^#^*p* < 0.05 vs. UN; ^&^*p* < 0.05 vs. UN2.5. The data were analyzed using a one-way analysis of variance followed by the Bonferroni post hoc test. Control (C, *n* = 9), undernutrition (UN, *n* = 5), undernutrition with 2.5 g/kg b.m. Chia oil supplementation (UN2.5, *n* = 8), and undernutrition with 5 g/kg b.m. Chia oil supplementation (UN5, *n* = 10).

**Figure 3:**
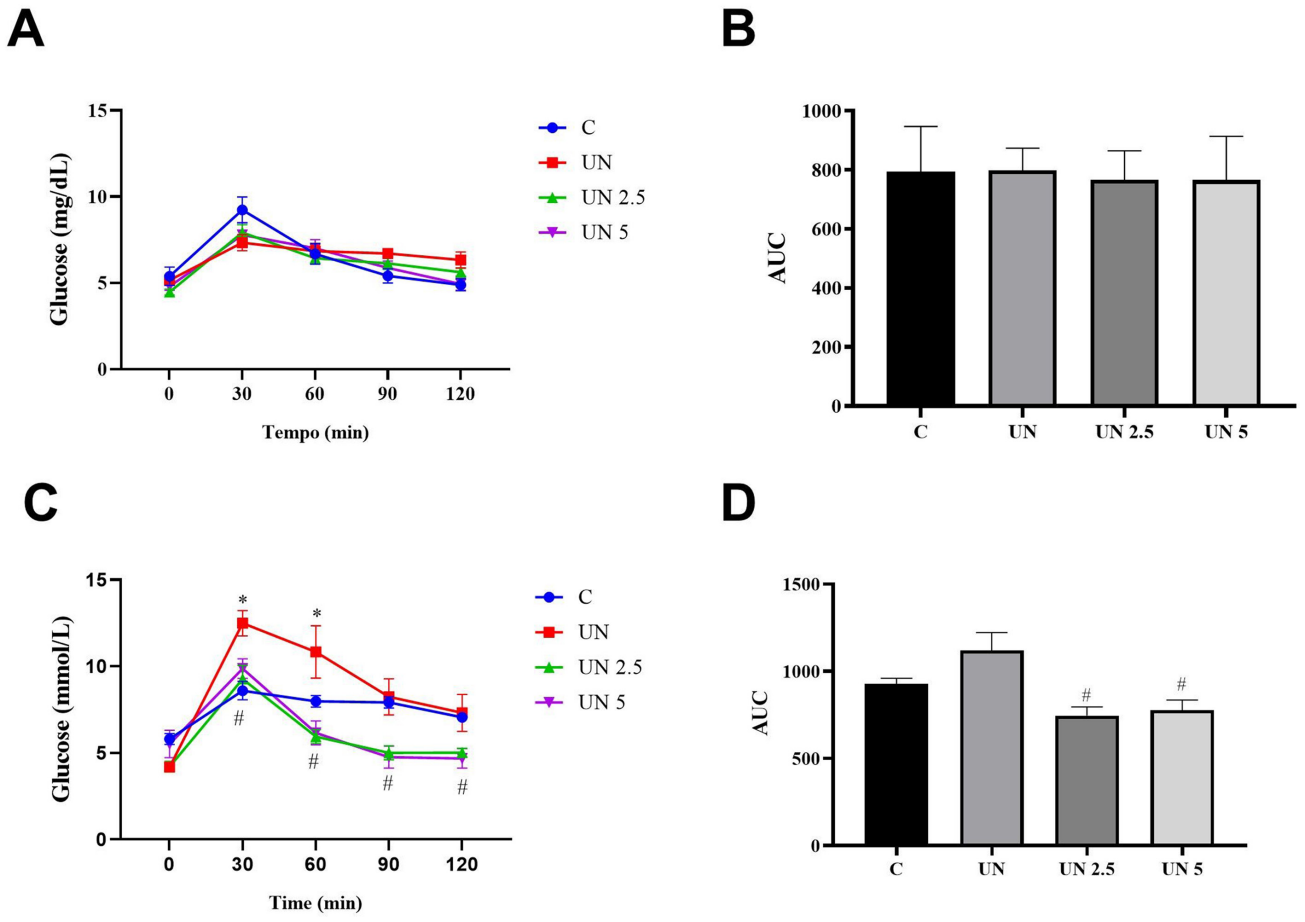
No significant differences in glycemic response were observed among the groups at 70 days **(A, B)**. The undernutrition litters from females supplemented with chia oil exhibited lower glycemic curves at 30, 60, 90, and 120 min **(C)** and lower glucose concentration at 120 days **(D)** compared to the undernutrition litter control. Data are presented as mean ± standard error of the mean. *p < 0.05 vs. C. ^#^*p* < 0.05 vs. UN. The data were analyzed using a one-way analysis of variance followed by the Bonferroni *post hoc* test. Control (C, *n* = 9), undernutrition (UN, *n* = 5), undernutrition with 2.5 g/kg b.m. Chia oil supplementation (UN2.5, *n* = 8), and undernutrition with 5 g/kg b.m. Chia oil supplementation (UN5, *n* = 10).

Glucose tolerance was evaluated at 70 and 120 days (Figure 3). No significant differences in glycemic response were observed among the groups at 70 days. At 120 days, however, the UN group showed higher blood glucose levels at 30 and 60 min than the control group (*p* < 0.05). The UN2.5 and UN5 groups displayed significantly lower glucose concentrations at 30, 60, 90, and 120 min than the UN group (*p* < 0.05). Moreover, these two supplemented groups had reduced glucose levels at 90 and 120 min relative to the controls (*p* < 0.05). The total glycemic exposure over time was also lower in the UN2.5 and UN5 groups than in the UN group at 120 days (*p* < 0.05).

**Figure 4:**
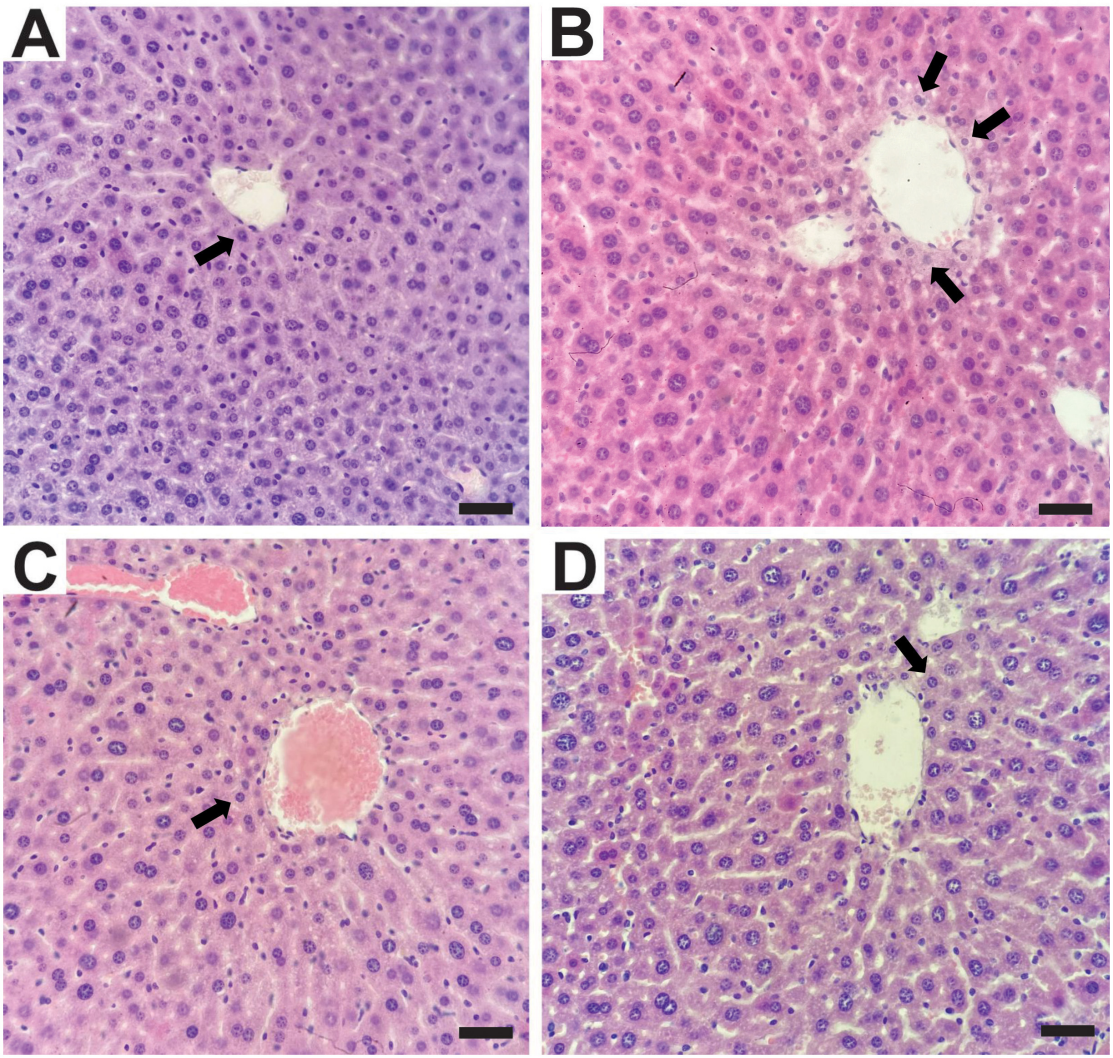
Histological sections of the liver were stained with hematoxylin and eosin (H&E). Central lobular region. **(A)** Control Group with multifocal areas of discrete vacuolization in the cytoplasm of hepatocytes (arrow); **(B)** undernutrition Group with moderate vacuolization in the cytoplasm of hepatocytes (arrows). **(C)** Undernutrition with 2.5 g/kg b.m. chia oil supplementation and **(D)** undernutrition with 5 g/kg b.m. chia oil supplementation with multifocal areas of discrete vacuolation in the cytoplasm of hepatocytes (arrow). Scale bars: 50 μm.

Histopathological analysis of liver tissue from animals in the experimental groups did not reveal significant alterations (Figure 4). The control group presented discrete microvacuoles in the hepatocyte cytoplasm in the centrilobular regions (Figure 4A), which is considered normal due to the liver's role in lipid metabolism. In contrast, the UN group exhibited multifocal areas with discrete to moderate cytoplasmic vacuolation in centrilobular hepatocytes (Figure 4B), which may be associated with food deprivation during the neonatal period and increased mobilization of triglycerides from adipose tissue (38). The UN2.5 and UN5 groups showed discrete microvacuoles similar to those observed in the control group (Figures 4C, D).

**Figure 5:**
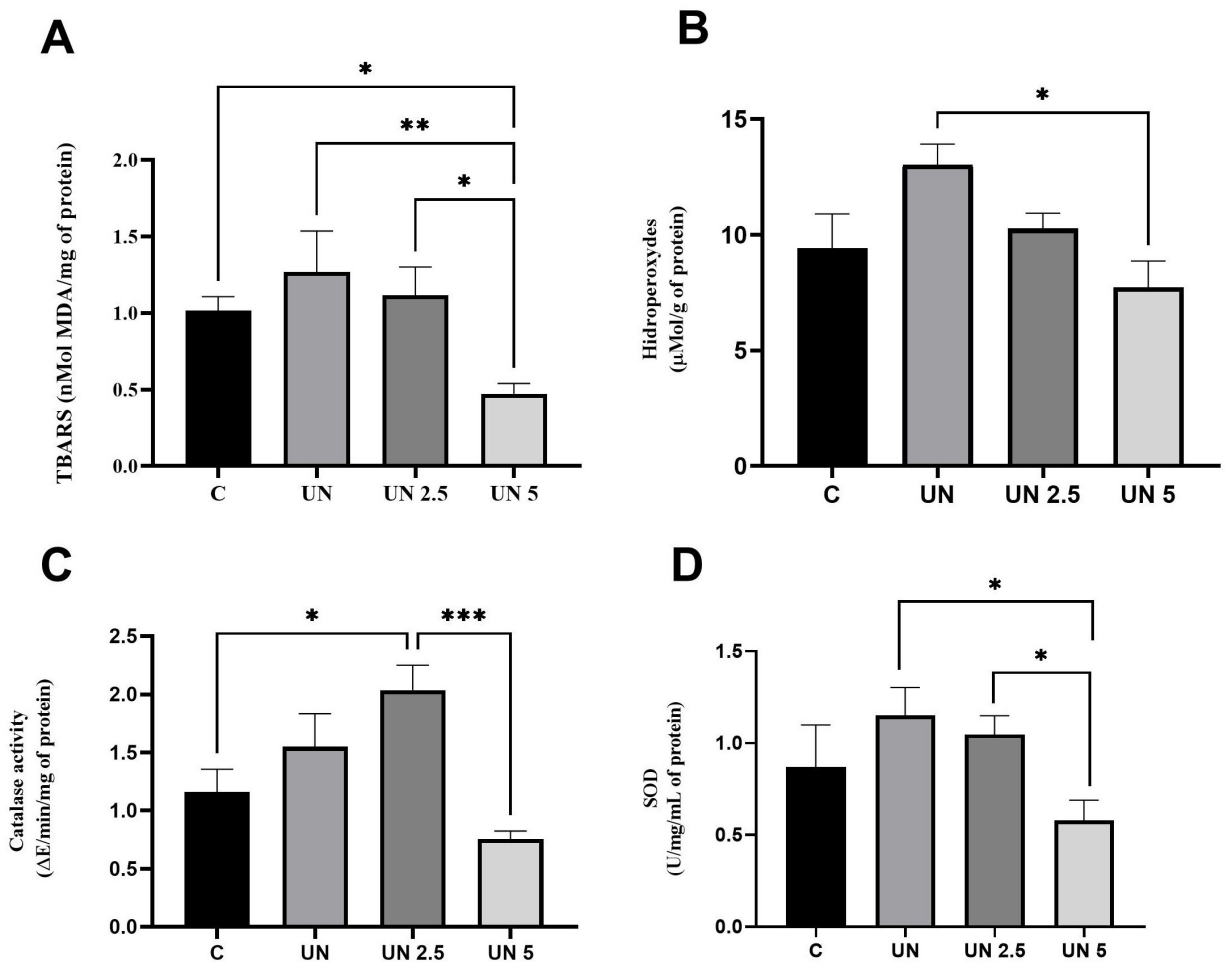
Maternal supplementation with chia oil influences the oxidant profile in the liver of undernourished offspring. The UN5 group exhibited lower TBARS and hydroperoxide levels **(A, B)**. The UN2.5 group exhibited higher CAT and SOD activities **(C, D)**. Data are presented as mean ± standard error of the mean. *p < 0.05; **p < 0.01; ***p < 0.001. The data were analyzed using a one-way analysis of variance followed by the Bonferroni *post hoc* test. Control (C, *n* = 9), Undernutrition (UN, *n* = 5), Undernutrition with 2.5 g/kg b.m. Chia oil supplementation (UN2.5, *n* = 8), and Undernutrition with 5 g/kg b.m. Chia oil supplementation (UN5, *n* = 10).

The UN5 group exhibited reduced TBARS levels in liver tissue compared to the Control (*p* = 0.04), UN (*p* = 0.007), and UN2.5 (*p* = 0.02) groups (Figure 5A). Liver hydroperoxide content (Figure 5B) was significantly lower in the UN5 group than in the UN group (*p* < 0.05). The UN2.5 group had higher catalase activity in the liver than the C (*p* = 0.02) and UN5 groups (*p* = 0.0001) (Figure 5C). Liver SOD activity was higher in the UN and UN2.5 groups than in the UN5 group (*p* < 0.05) (Figure 5D).

**Figure 6:**
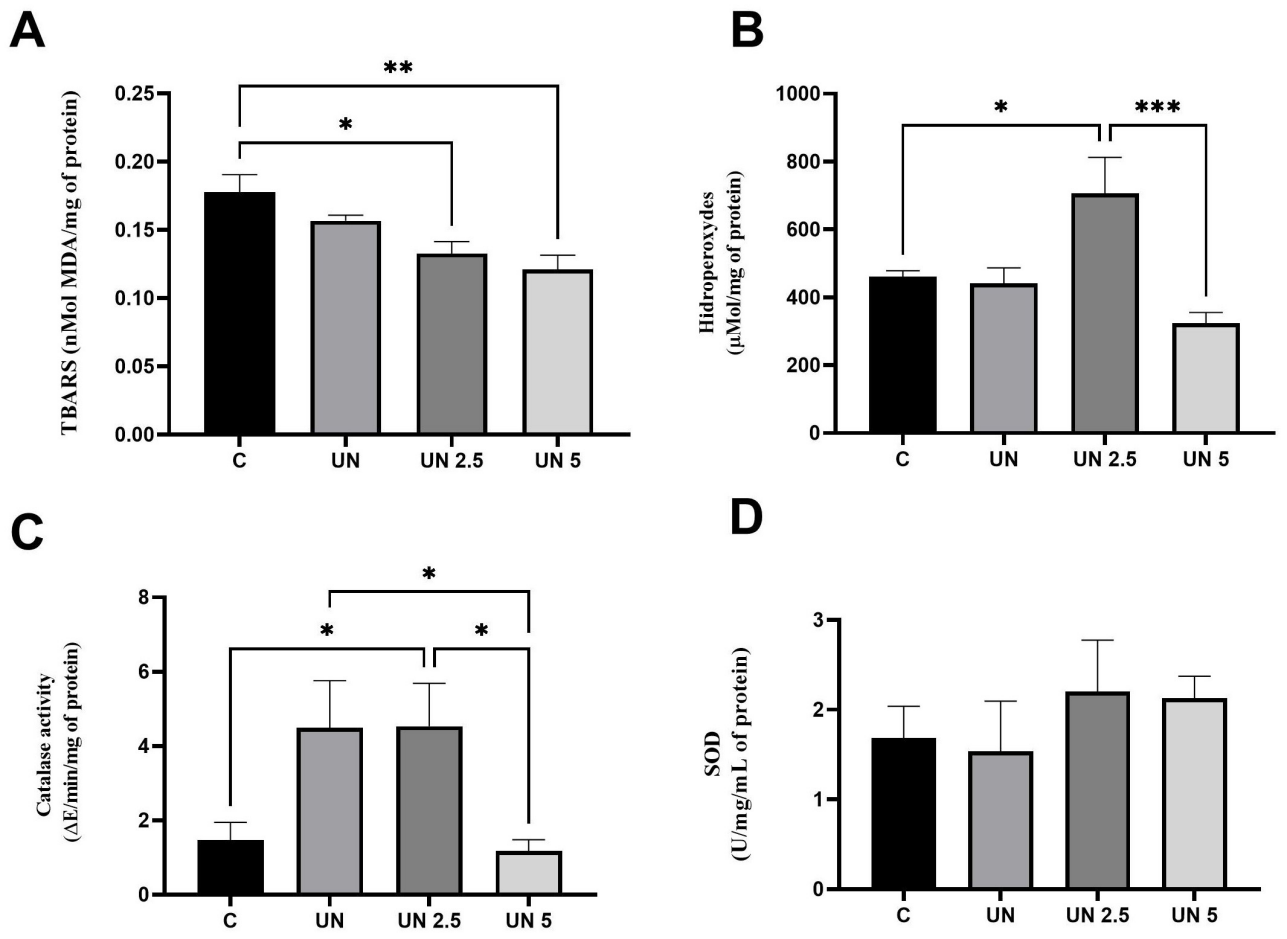
Maternal chia oil supplementation affects the oxidant profile of undernourished offspring in the epididymal adipose tissue. The UN2.5 and UN5 litters exhibited lower TBARS concentrations **(A)**. UN 2.5 exhibited higher hydroperoxide levels, while UN 5 showed lower levels **(B)**. Catalase activity was higher in the UN2.5 group **(C)**. No significant changes were observed in SOD activity **(D)**. Data are presented as mean ± standard error of the mean. *p < 0.05; **p < 0.01; ***p < 0.001. The data were analyzed using a one-way analysis of variance followed by the Bonferroni *post hoc* test. Control (C, *n* = 9), Undernutrition (UN, *n* = 5), Undernutrition with 2.5 g/kg b.m. Chia oil supplementation (UN2.5, *n* = 8), and Undernutrition with 5 g/kg b.m. Chia oil supplementation (UN5, *n* = 10).

Oxidative stress markers were also evaluated in the epididymal adipose tissue (Figure 6). TBARS levels were significantly decreased in the UN2.5 (*p* = 0.03) and UN5 (*p* = 0.003) groups compared to the controls (Figure 6A). Hydroperoxide concentrations were elevated in the UN2.5 group compared to those in the Control, UN, and UN5 groups (*p* < 0.05) (Figure 6B). CAT activity was higher in the UN2.5 group than in the control group (*p* < 0.05), whereas the UN5 group showed reduced CAT activity compared to the UN group (*p* = 0.005) (Figure 6C). No significant differences were observed in SOD activity in the liver tissue across the groups (Figure 6D).

## 4 Discussion

Our results demonstrate that maternal supplementation with ChO, a rich source of ALA, effectively improved glucose tolerance and redox homeostasis in offspring subjected to postnatal undernutrition during lactation. The ALA content in ChO may be the key bioactive component responsible for improvements in glucose metabolism and insulin sensitivity. However, its concentration can vary depending on the seed variety and environmental factors such as temperature, climate, and soil conditions (39, 40). In the present study, gas chromatography analysis revealed that ALA accounted for 52.3% of the total fatty acid content in ChO. Based on this, the dose of 5 g/kg corresponds to 2.61 g of ALA, while the dose of 2.5 g/kg corresponds to 1.31 g/day.

We did not find any significant alterations in the histopathological analysis. In fact, ChO has been linked with the amelioration of hepatic disturbances in rats fed a high-fat and high fructose diet (41). It is important to note that our analysis was limited to hematoxylin and eosin staining, which may not reveal more subtle morphological changes. Despite the absence of clear morphological changes, functional metabolic outcomes were positively affected by maternal ChO supplementation. We observed an improvement in glucose tolerance, as the UN2.5 and UN5 groups exhibited reduced AUC at 120 days compared to the UN group, even though the UN5 group had greater fasting glucose levels than the Control group. Undernourished animals exhibit normal glucose tolerance (42, 43), despite a reduction in glucose-stimulated insulin secretion. Previous studies have shown that 3g ALA supplementation improves insulin sensitivity and glucose tolerance, but fasting glucose levels may be influenced by other factors, such as hepatic metabolism (44, 45).

ChO has been shown to positively regulate the transcript levels of insulin receptors. Additionally, the phenolic compounds present in ChO may aid in regulating glucose levels by inhibiting gluconeogenesis (46). The supplementation with 10% flaxseed oil, another source of ALA, also led to a significant increase in hepatic mRNA expression of PPAR-γ, suggesting that ALA may activate the PPAR-γ dependent pathway to alter liver lipid metabolism and enhance insulin sensitivity (47). Souza et al. (48) observed improved glucose responses in Swiss mice fed a high-fat diet and supplemented with ChO in a dose of 1.5g/kg b.m. for 6 weeks. Poudyal et al. (45) supplemented Wistar rats with ChO at 30ml/kg of diet for 8 weeks and found improvements in glucose tolerance and insulin sensitivity without changes in lipid plasma.

Maternal fatty acids are believed to be transported to the fetus via specific transmembrane proteins: fatty acid transport proteins (FATPs), fatty acid translocase (FAT/CD36), and intracellular fatty acid-binding proteins (FABPs). Once fatty acids enter the cells surrounding the fetus, they can be translocated to the nucleus to modulate gene expression, stored for later use, or directed to the mitochondria to regulate mitochondrial function (15–17). Maternal dietary enrichment with ω-3 PUFAs is linked to increased ω-3 PUFA accumulation in the offspring liver (47), which increases the activity of key mitochondrial enzymes, including citrate synthase, isocitrate dehydrogenase, and α-ketoglutarate dehydrogenase. These enzymes are essential components of the tricarboxylic acid cycle, and their enhanced activity suggests improved mitochondrial efficiency. By supporting mitochondrial function, ω-3 PUFA intake during the perinatal period may help prevent insulin resistance via glycemic control. Another suggested mechanism is that maternal ω-3 supplementation modulates offspring glucose metabolism by upregulating genes involved in fatty acid oxidation (e.g., carnitine palmitoyltransferase I and acyl-CoA oxidase 1) and glycolysis/gluconeogenesis (e.g., glycerol-3-phosphate dehydrogenase 1), while downregulating genes responsible for fatty acid synthesis (e.g., ATP-citrate lyase and stearoyl-CoA desaturase 1). This gene expression profile promotes greater triglyceride catabolism and reduces hepatic lipid synthesis in offspring. Moreover, ω-3 has been shown to decrease pyruvate kinase activity, suggesting a decrease in glucose oxidation as fatty acid oxidation increases. This highlights the central role of ω-3 fatty acids in regulating fatty acids and glucose in neonates (49, 50).

In addition to these lipid-regulating effects, ω-3 PUFAs have also been shown to mitigate oxidative stress through multiple mechanisms. Indeed, prior investigations have demonstrated that ω-3 supplementation reduces malondialdehyde (MDA) levels, an end product of lipid peroxidation and an established oxidative stress marker (51). This effect may occur through ω-3-mediated modulation of prostaglandin composition, as ω-3 acts as a potent inhibitor of the arachidonic acid prostaglandin production pathway, which is known for its pro-inflammatory properties. Additionally, ω-3 has been shown to inhibit cyclooxygenase-2 (COX-2) enzyme activity (39, 40, 52–55), potentially explaining its MDA-lowering effects, as COX-2 generates oxidative and inflammatory prostaglandins that promote lipid peroxidation.

The UN2.5 group exhibited higher CAT activity in both tissues and greater hydroperoxide levels in the epididymal tissue. Earlier studies have established that hydroperoxides are absent in normal plasma owing to degradative systems such as catalase (56). Thus, the increase in catalase activity suggests an attempt to reduce the hydroperoxides, which are increased in conditions of oxidative stress, as in the undernutrition model used in our study. Consistent with these findings, Rincón-Cervera et al. (57) provided 21 days of chia oil (100 g/kg of diet) and observed high antioxidant enzyme (SOD, CAT, glutathione peroxidase, and glutathione reductase) activity in the liver. Other studies have shown the enhancement of antioxidant enzymes and improvement in oxidative stress parameters through ChO supplementation. It described the improvement of antioxidant activity in plasma and liver, from several doses of ChO (1.61 g/mL, 4.28 g/mL, 4.22 g/ml) (41, 51, 58, 59). Moreover, previous studies have suggested that ω-3 fatty acids may stimulate the NF-κB pathway via activation of PPAR-α, a mechanism that regulates the expression of several antioxidant enzymes, a previous work by Han et al. (51), showed that a supplementation of 3,8 g/kg b.m. upregulated the PPAR-α. The non-enzymatic peroxidation of ω-3 generates a product called J3-isoprostanes, which leads to the expression of several antioxidant enzymes in the liver, formation of glutathione, and a decrease in lipid peroxidation rates (60, 61).

Moreover, ChO is a source of bioactive compounds due to its high content of polyphenolic compounds (e.g., chlorogenic acid, caffeic acid, myricetin, quercetin, and kaempferol) (62). The antioxidant activity of these polyphenols involves multiple mechanisms, including reactive oxygen species (ROS) scavenging, suppression of ROS generation by inhibiting specific enzymes, chelation of trace elements, and upregulation of endogenous antioxidant defenses (63). Some studies have demonstrated that polyphenols can reduce oxidative stress markers without significant changes in antioxidant enzyme activity, suggesting a direct antioxidant effect (63, 64). The UN5 group showed reduced oxidative stress biomarkers (TBARS and hydroperoxides), along with lower activity of antioxidant enzymes (CAT and SOD). In contrast, the UN2.5 group presented higher levels of hydroperoxides in adipose tissue and increased activity of antioxidant enzymes in both analyzed tissues. These findings suggest a dose-dependent effect on oxidative stress. The higher dose (UN5) may exert antioxidant effects through mechanisms independent of antioxidant enzymes, possibly by directly scavenging ROS or suppressing their generation via inhibition of specific enzymes, thus reducing the need for upregulation of endogenous antioxidant defenses. On the other hand, the 2.5 g/kg dose appears to induce the activation of antioxidant enzymes as a compensatory response to oxidative stress.

Our findings indicate that postnatal undernutrition leads to reduced body weight during the early stages of development. Triglycerides are the predominant lipids in milk. Throughout the suckling period, their concentration tends to decline, and in large litters, competition among pups further reduces individual milk intake. Kozak et al. (65) have demonstrated that undernourished animals have smaller adipocytes, indicating reduced lipid accumulation. In rodents, most white adipose tissue development occurs during the postnatal period, particularly during lactation, although some adipogenesis begins prenatally. Despite their higher weight gain, undernourished offspring did not reach the body mass of the Control group up to 120 days of life, which aligns with previous findings (8, 9, 25, 42). Previous studies have found alterations in body composition, such as fat redistribution, decreased fat mass, and increased lean mass, in animals supplemented with ALA derived from chia oil (45, 66–68). Additionally, maternal dietetic supplementation with ALA (7g of linseed oil/100g of diet) (69) has been linked with lower fat accumulation, which is seen both in lower fat mass and in reduced adipocyte size in offspring.

ALA intake may be associated with reduced body adiposity due to a lipid redistribution with FAT/CD36 (fatty acid translocase/cluster of differentiation 36) recruitment to the plasma membrane, mitochondrial activity, and beta-oxidation. The PUFAs n-3 positively regulate lipoprotein lipase and adipose triacylglycerol lipase, which play important roles in catalyzing the hydrolysis of triacylglycerol into fatty acids and monoacylglycerol in lipoproteins, skeletal muscle, and adipose tissue. However, the exact mechanism by which ALA exerts its effects remains unclear. Owing to the differences in pharmacological response to ALA and to EPA/DHA, ALA may have effects through alternative mechanisms that require further investigation (45, 46, 59, 70). ALA supplementation at 4 g/day in obese subjects reduced free fatty acids and increased a gene that inhibits adipose triglyceride lipase (G0S2) and PPAR-γ expression in peripheral blood mononuclear cell, indicating an antilipolytic effect (71). The upregulation of G0S2, may be beneficial in preventing excessive lipolysis and the consequent release of free fatty acids into circulation, which is associated with insulin resistance and metabolic dysfunction. In this context, reducing lipolysis could help preserve metabolic homeostasis, particularly under conditions of early-life nutritional stress (72). Another study supplementing 3g of ALA showed a significant serum reduction in IL-6, IL-1β and chemoattractant protein-1 (MCP-1), suggesting that ALA supplementation prevents the inflammatory process in animals fed a high-fat diet (73).

Our results suggest that the influence of ChO is dose-dependent, where a dose of 5 g/kg, in parallel to a decrease in fat mass, attenuated the total weight gain when compared to the offspring from mothers supplemented with the dose of 2.5 g/kg. These findings may be partly explained by ALA's capacity to alter plasma lipid composition and metabolism. Indeed, prior evidence has demonstrated that 15–30 days of supplementation with 10%−24% ALA in dietary lipids is sufficient to increase levels of ALA and EPA in the bloodstream; in this way, the enrichment of plasma lipids by the consumption of ALA influences the type of lipoproteins synthesized by the liver (very-low-density lipoprotein, high-density lipoprotein, low-density lipoprotein) and the peripheral distribution of ALA by increasing the bioaccessibility of PUFA ω-3 in the body (74). It has also been described that the higher the dietary ALA content, the higher the hepatic conversion of EPA and DHA (57).

Although our study suggests that maternal supplementation with ChO may induce long-term metabolic and redox improvements in offspring, we did not directly assess epigenetic mechanisms, which were hypothesized to underlie these effects. Therefore, the involvement of epigenetic modulation remains speculative and should be interpreted with caution. In addition, the study was conducted exclusively in male offspring, which limits the generalizability of our findings. Given that sex-specific responses to nutritional and oxidative challenges have been reported, future investigations involving both sexes are necessary to provide a more comprehensive understanding of the observed effects. Further studies incorporating epigenomic and transcriptomic approaches are warranted to confirm the potential molecular mechanisms involved. Also, future studies employing complementary staining techniques, such as Masson's trichrome, Oil Red O, or PAS, are warranted to provide a more detailed evaluation of liver structure, including fibrosis, lipid accumulation, and glycogen content.

Based on our findings, maternal supplementation with chia oil (ChO) attenuated several metabolic disturbances associated with early-life undernutrition. Specifically, supplementation improved parameters such as compensatory weight gain, glucose tolerance, hepatic and epididymal levels of TBARS and hydroperoxides, and hepatic catalase (CAT) activity. The lower dose (2.5 g/kg b.m.) was associated with improvements in glucose tolerance and CAT activity, while the higher dose (5 g/kg b.m.) more effectively reduced compensatory weight gain, improved glucose handling, lowered oxidative stress markers, despite no observed upregulation of antioxidant enzyme activity, and decreased hepatic lipid accumulation. These dose-dependent effects indicate a modest advantage of a higher dose in mitigating long-term metabolic alterations. However, the precise mechanisms, particularly those related to the role of ALA in early metabolic programming, remain unclear. Further studies are needed to clarify these effects and to determine the translational relevance of maternal ALA-rich supplementation in undernutrition settings.

## Data Availability

The raw data supporting the conclusions of this article will be made available by the authors, without undue reservation.
